# Infection of post-harvest peaches by *Monilinia fructicola* accelerates sucrose decomposition and stimulates the Embden–Meyerhof–Parnas pathway

**DOI:** 10.1038/s41438-018-0046-x

**Published:** 2018-09-01

**Authors:** Jingyu Kou, Yingying Wei, Xingxing He, Jiayu Xu, Feng Xu, Xingfeng Shao

**Affiliations:** Department of Food Science and Engineering, Ningbo University, Ningbo, China

## Abstract

To study the changes in sugar metabolism caused by fungal infection in post-harvest peaches, fruit from two cultivars (‘Baifeng’ and ‘Yulu’) was inoculated with *Monilinia fructicola* and stored at 10 °C. During disease development, soluble sugar content was monitored, as well as the activities and expression of selected enzymes. Disease progression was accompanied by a decrease in sucrose content and increases in reducing sugars and soluble solids, consistent with higher enzyme activities for acid invertase, neutral invertase and sucrose synthase-cleavage, and lower activities for sucrose synthase-synthesis and sucrose phosphate synthase. Activities of phosphofructokinase, hexokinase, and pyruvate kinase, which are related to hexose metabolism, also increased. These changes stimulate the Embden–Meyerhof–Parnas (EMP) pathway. We conclude that the fungal disease in peach fruit accelerates the decomposition of sucrose, thereby providing more glucose as a substrate to the EMP pathway.

## Fruit fungal infection: Effects on sugar metabolism

A study by researchers in China provides insights into changes to sugar metabolism during fungal infection in post-harvest peaches. Fungal infections and associated disease development can alter sugar metabolism in post-harvest fruits, leading to rapid decay and a short shelf life. However, little is known about the mechanisms behind these fungal infections. Xingfeng Shao and Yingying Wei at Ningbo University and co-workers examined the effect of brown rot, caused by a fungus called *Monilinia fructicola*, on sugar metabolism in two peach cultivars kept under chilled conditions. As the disease progressed, the fruit increased its energy supply by decomposing sucrose and generating more glucose. The team uncovered the major enzymes responsible for this sucrose decomposition. The increased glucose stimulated the Embden-Meyerhof-Parnas pathway, which in turn helped the peaches fight infection.

## Introduction

Soluble sugars including sucrose, glucose and fructose, play an important role in biotic and abiotic stress in plants. Abiotic stress such as drought, high temperature, and low temperature can stimulate the accumulation of reactive oxygen species (ROS) that cause extensive damage to the cell. Sugars can act as important roles in the response to abiotic oxidative stress, including ROS scavenger^1^, regulate osmotic pressure, and protect cell membranes^2^, signaling molecules to regulate gene expressions^3^. Many studies concerning soluble sugars have been conducted in fruits during cold storage, which showed that sugar content directly affects post-harvest cold resistance in peaches^4^, apricots^5^, and loquat fruit^6^. Meanwhile, benzothiadiazole treatment altered soluble sugar components and enhanced disease defense-related gene expression and phytoalexin accumulation in grape^7^. The increased concentrations of soluble sugars in grape berries, were accompanied by abundance of antifungal proteins, showing a close link between sugar metabolism and disease resistance^8^.

Fungal infection and disease development, as a common biotic stress in plants, can also lead to a change in sugar metabolism. Peach leaves suffering from peach leaf curl, a disease caused by the ascomycetous fungus *Taphrina deformans* (Berk.), have higher levels of fructose and especially glucose, but are reduced in sucrose and sorbitol content^9^. In peach fruit, fungal disease is the major factor that causes post-harvest decay and reduces the shelf life^10^. Sucrose, which is the most abundant sugar in peach fruit^11^, degrades during the ripening process, while levels of reducing sugars (glucose and fructose) increase^12^. Acid invertase (AI), neutral invertase (NI), and sucrose synthase-cleavage (SS-cleavage) are the major enzymes responsible for sucrose decomposition^13^. Sucrose synthesis is catalyzed by synthase-synthesis (SS-synthesis) and sucrose phosphate synthase (SPS)^14^. Our previous studies on peach fruit demonstrated that low temperature stress causes a sharp decline in sucrose levels due to increased NI and AI activities^15^, if higher sucrose concentrations can be maintained, peaches are more tolerant to chilling injury^16^.

Brown rot caused by *Monilinia fructicola* is the primary disease affecting harvested peach fruit. Changes in sucrose metabolism that occur when peaches are stressed by invading fungi have not been investigated previously. In the study reported here, peaches from two cultivars were inoculated with *M. fructicola* and stored at 10 °C to observe changes in soluble sugar content, soluble sugar metabolism-related enzyme activity, and gene expression.

## Materials and methods

### Fruit and fungal inoculation

*M. fructicola* was isolated from an infected peach fruit and identified by the China General Microbiological Culture Collection Center. It’s cultured on potato dextrose agar (PDA: 1 L deionized water in which 200 g potatoes have been boiled, plus 20 g glucose, and 20 g agar) at 25 °C. Spores from a one-week-old culture were suspended in 0.9% (m/v) NaCl, and the spore concentration was adjusted to 1 × 10^5^ mL^−1^ using a hemocytometer.

‘Baifeng’ and ‘Yulu’ peach fruit (*Prunus persica* L. Batsch) were harvested at commercial maturity in Zhejiang Province, China, and selected for uniform size, the absence of physical injuries, and absence of disease. Prior to treatment, fruit were placed at 5 °C for 6 h to remove field heat. A total of 600 peach fruit of each cultivar were used. Fruits were wounded once to a depth of 4 mm with a sterilized nail (3 mm diameter) in the equatorial zone, and were randomly assigned to a control group (fruit directly stored at 10 °C) or an inoculated group (fruit inoculated with 20 µL 1 × 10^5^ mL^−1^ of *M. fructicola* spore suspension and then stored at 10 °C). ‘Baifeng’ and ‘Yulu’ fruit were stored for 20 d and 16 d, respectively. Disease incidence and lesion diameter were measured every 4 d for ‘Yulu’ and every 5 d for ‘Baifeng’. Healthy tissue samples surrounding each wound (10 mm distance from the edge of the wound, 10 mm height from the peel) were collected, frozen in liquid nitrogen, and stored at −80 °C. Three replicates consistent of 10 fruit were sampled at each time point in both inoculated group and control group, and the experiments were conducted twice.

### Determination of decay development, soluble solids content (SSC), and soluble sugars content

Disease incidence was calculated as the percentage of fruit displaying rot, while lesion diameter was measured using the cross method for infected wounds. Soluble solids content was determined by analyzing fruit juice with digital refractometer (PAL-1, Atago, Japan). Soluble sugars content was measured according to the method of Yu et al.^17^, and results expressed as milligrams per gram fresh weight (FW).

### Enzyme activities related to sucrose metabolism and hexose metabolism

Frozen samples (1 g) were homogenized in 5 mL of 100 mM sodium phosphate buffer (pH = 7.5) containing 5 mM MgCl_2_, 1 mM ethylenediaminetetraacetic acid, 2.5 mM dithiothreitol, and 0.1% (v/v) Triton X-100, using a cold mortar and pestle. The homogenates were centrifuged at 10,000× *g* for 20 min at 4 °C. The supernatant was transferred to a dialysis bag (MD 10-14-5) and placed in a 50-fold volume of extraction buffer (without Triton X-100) at 4 °C for one night. Dialyzed samples were used for enzyme activity measurements as described below.

Activities of enzymes related to sucrose metabolism (AI, NI, SS-cleavage, SS-synthesis, and SPS) were measured and calculated according to the method of Yu et al.^17^ One unit of AI, NI and SS-cleavage activities were defined as the amount of enzyme that catalyzes the creation of 1 μmol of glucose per minute. One unit of SS-synthesis and SPS activities were defined as the amount of enzyme that synthetize 1 μmol of sucrose per minute. For hexose metabolism, activities of glucose 6-phosphate dehydrogenase (G6PDH), phosphofructokinase (PFK), hexokinase (HXK), and pyruvate kinase (PK) were measured using the corresponding Plant ELISA Kit (Yuanye, Shanghai, China) according to the manufacturer’s instructions. One unit of G6PDH activity was defined as the amount of enzyme that produces 1 nmol of nicotinamide adenine dinucleotide phosphate per minute. One unit of PFK activity was defined as the amount of enzyme that catalyzes 1 nmol of fructose 6-phosphate to produce fructose 1, 6-diphosphate per minute. HXK activity was measured as the total glucose-phosphorylating capacity. One unit of PK activity was defined as the reduction of 1 nmol of nicotinamide adenine dinucleotide per minute. All these enzyme activities were expressed as U/g FW.

### RNA isolation and real-time quantitative PCR (qPCR) analysis

Total RNA was isolated from frozen tissues samples using the RNAplant Plus Reagent (Tiangen, Beijing, China) according to the manufacturer’s instructions. RNA was quantified using a spectrophotometer (NanoDrop 2000, Thermo, USA) and tested for integrity by agarose electrophoresis. cDNAs were synthesized using SuperMix for qPCR (Vazyme, Nanjing, China) following the provided instructions. The cDNA was diluted 30-fold, and 4 µL of the diluted cDNA was used as the template for qPCR analysis. The reaction mix (20 µL final volume) consisted of 10 µL of Master Mix (Vazyme, Nanjing, China), 0.8 µL each primer (10 µM), 0.4 µL 50 × ROX Reference Dye 2 (Vazyme, Nanjing, China), 5 µL cDNA, and 3.6 µL RNase-free water. The thermocycling program consisted of an initial hold at 95 °C 30 s, followed by 40 cycles of 10 s at 95 °C, and 30 s at 60 °C. All qPCR reactions were normalized by the threshold cycle value (Ct) compared to a housekeeping gene TEF2 (GeneBank Accession: JQ732180) following the 2^−∆∆Ct^ method for relative quantification^18^. Each RNA sample was determined as an average three independent experiments. Primers were designed on the peach nucleotide sequence at National Center for Biotechnology Information. The sequences of primers were as follow: *HXK*1 (GeneBank Accession: AF367451.1, forward: 5′-AGATGTGGTGGGAGAGCTGA-3′; reverse 5′-ATGGCATGAGCCCGTTCTAC-3′) and *HXK2* (GeneBank Accession: AF367452.1, forward: 5′-CCTGGCAGGCAGAGAGAAC-3′; reverse: 5′-TAACCAGAGCAGACACACGC-3′).

### Statistical analysis

The data were expressed as means ± standard error (SE). Statistical analysis was performed using the SAS package program version 8.0 (SAS Institute, Cary, NC, USA). Main effects were analyzed and means were compared by Duncan’s multiple range tests at a significance level of 0.01 or 0.05.

## Results

### The development of disease in peach fruit

Disease incidence in inoculated ‘Baifeng’ fruit reached 86.7 and 100% after storage at 10 °C for 5 d and 10 d, respectively (Table 1). For inoculated ‘Yulu’fruit, disease incidence was 80 and 100% after storage at 10 °C for 8 d and 12 d. Lesion diameters increased with the storage time, and reached 9.32 cm (‘Baifeng’) and 9.12 cm (‘Yulu’) by the end of storage. Peaches inoculated with *M. fructicola* developed particularly severe decay.

**Table 1:** The development of disease caused by *M. fructicola* in ‘Baifeng’ and ‘Yulu’ peach fruit

Baifeng			Yulu		
Storage time	Disease incidence (%)	Lesion diameter(cm)	Storage time	Disease incidence (%)	Lesion diameter(cm)
Day 5	86.7 ± 6.7	1.06 ± 0.01	Day 4	0	0
Day 10	100	4.01 ± 0.02	Day 8	80 ± 10.0	2.42 ± 0.14
Day 15	100	8.32 ± 0.07	Day 12	100	4.90 ± 0.17
Day 20	100	9.32 ± 0.07	Day 16	100	9.12 ± 0.16

Data are expressed as the mean ± SE (*n* = 6)

### Changes in soluble sugar content and SSC with development of fungal disease

**Fig. 1:**
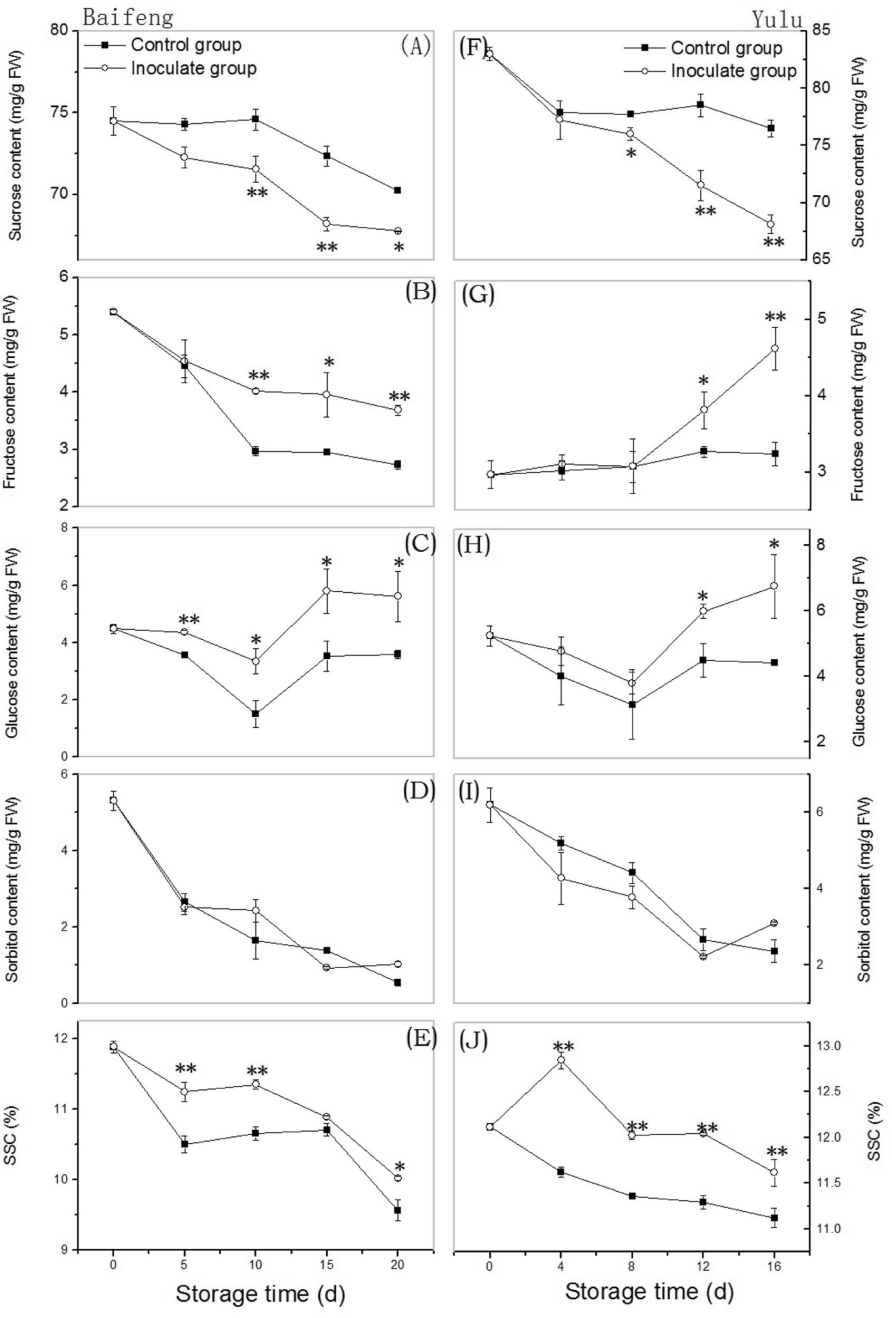
Changes in soluble sugars content and SSC in ‘Baifeng’ (A-E) and ‘Yulu’ (F-J) peaches stored 10 °C for 20 and 16 d, respectively. Data are expressed as the mean ± SE (*n* = 6). Asterisks indicate significant differences between control and inoculated fruit at each time point (*p < 0.05; **p < 0.01 based on Duncan’s multiple range test)

As shown in Fig. 1, disease progression in ‘Baifeng’ and ‘Yulu’ fruit was accompanied by significant changes in sucrose, fructose, and glucose content. In both cultivars, sucrose content declined relative to controls (Fig. 1a, f) while inoculated fruit reached significantly higher levels of the reducing sugars fructose (Fig. 1b, g) and glucose (Fig. 1c, h) at later stages. No significant (*p* > 0.05) differences in sorbitol content between inoculated and control fruit were observed (Fig. 1d, i). However, the infected fruit had significantly (*p* < 0.05 for ‘Baifeng’, *p* < 0.01 for ‘Yulu’) higher SSC during storage (Fig. 1e, j). These results indicate that sucrose is lost more rapidly in the presence of fungal disease, while reducing sugar content and SSC increase.

### Changes in activities of sucrose-metabolizing enzymes during development of fungal disease

**Fig. 2:**
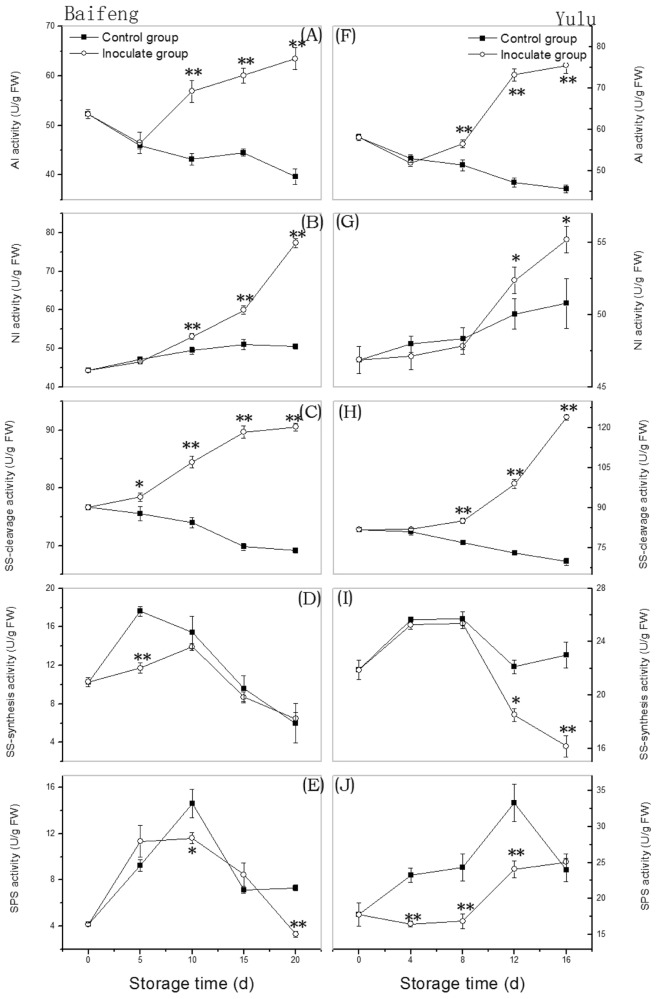
Changes in activity of sucrose-metabolism enzymes in ‘Baifeng’ (A-E) and ‘Yulu’ (F-J) peaches stored at 10 °C for 20 and 16 d, respectively. Data are expressed as the mean ± SE (*n* = 6). Asterisks indicate significant differences between control and inoculated group at each time point (*p < 0.05; **p < 0.01 based on Duncan’s multiple range test)

Activities for some key enzymes involved in sucrose metabolism also changed as a result of infection. After a short lag, AI activity (Fig. 2a, f) increased in both cultivars after infection, but declined in controls. Increases were also observed in infected fruit for NI (Fig. 2b, g) and SS-cleavage activity (Fig. 2c, h). For NI, activity increased in the controls as well, but not as rapidly, while for SS-cleavage activity, controls showed a steady decline.

SS-synthesis profiles were qualitatively similar for cultivars and controls, increasing initially and then decreasing (Fig. 2d, i). However, divergence between infected and uninfected ‘Baifeng’ fruit was significant only on day 5. In contrast, for ‘Yulu’, SS-synthesis activity for inoculated fruit was significantly (*p* < 0.05) lower than in controls on days 12 and 16. SPS activity for infected ‘Baifeng’ peaches was significantly (*p* < 0.05) lower than in controls at 10 d and 20 d (Fig. 2e). However, for ‘Yulu’, SPS activity was lower than in controls lower than control group except at the beginning and end of the experiment (Fig. 2j). Overall, these results show that activities for enzymes involved in sucrose cleavage increase as decay progresses, but activities of enzymes related to sucrose synthesis decrease.

### Activities of hexose-metabolizing enzymes during development of fungal disease

**Fig. 3:**
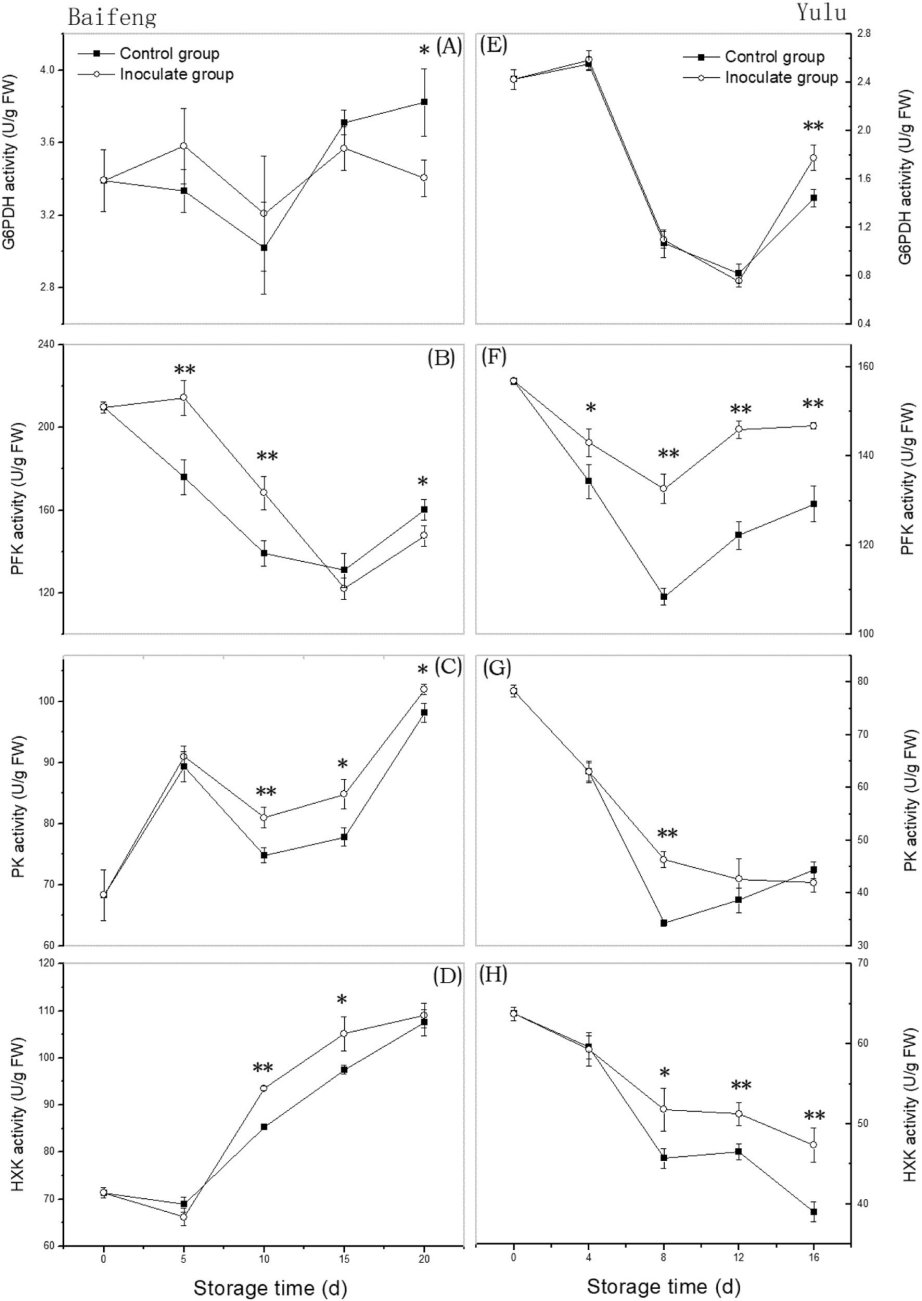
Changes in activity of hexose-metabolism enzymes in ‘Baifeng’ (A-D) and ‘Yulu’ (E-H) peaches stored at 10 °C for 20 and 16 d, respectively. Data are expressed as the mean ± SE (*n* = 6). Asterisks indicate significant differences between control and inoculated group at each time point (*p < 0.05; **p < 0.01 based on Duncan’s multiple range test)

In infected and uninfected ‘Baifeng’ and ‘Yulu’ peaches, G6PDH activity decreases and then increases (Fig. 3a, e). Only on the last day of sampling do infected and uninfected fruit differ significantly, with infected fruit showing either lower (‘Baifeng’) or higher (‘Yulu’) G6PDH activities. PFK (Fig. 3b, f) activity also decreases at first and then increases in control and diseased fruit. Activity is consistently higher in inoculated fruit except for ‘Baifeng’ on day 15. ‘Baifeng’ and ‘Yulu’ cultivars have distinct PK and HXK activity profiles. In ‘Baifeng’ peaches, activities for both enzymes generally rise (Fig. 3c, d), but in ‘Yulu’ they decline (Fig. 3g, h). Fungal disease appears to increase PK and HXK activities in ‘Baifeng’ peaches after 5 d and 10–15 d of storage, respectively. This is also true for PK activity in diseased ‘Yulu’ fruit, but the difference is significant only on day 8. Finally, HXK activity is significantly higher in diseased fruit for ‘Baifeng’ (days 10 and 15) and ‘Yulu’ (days 8, 12, and 16). These results reveal that PFK, PK, and HXK activities are increased in decaying peaches.

### Transcription of HXK genes during development of fungal disease

**Fig. 4:**
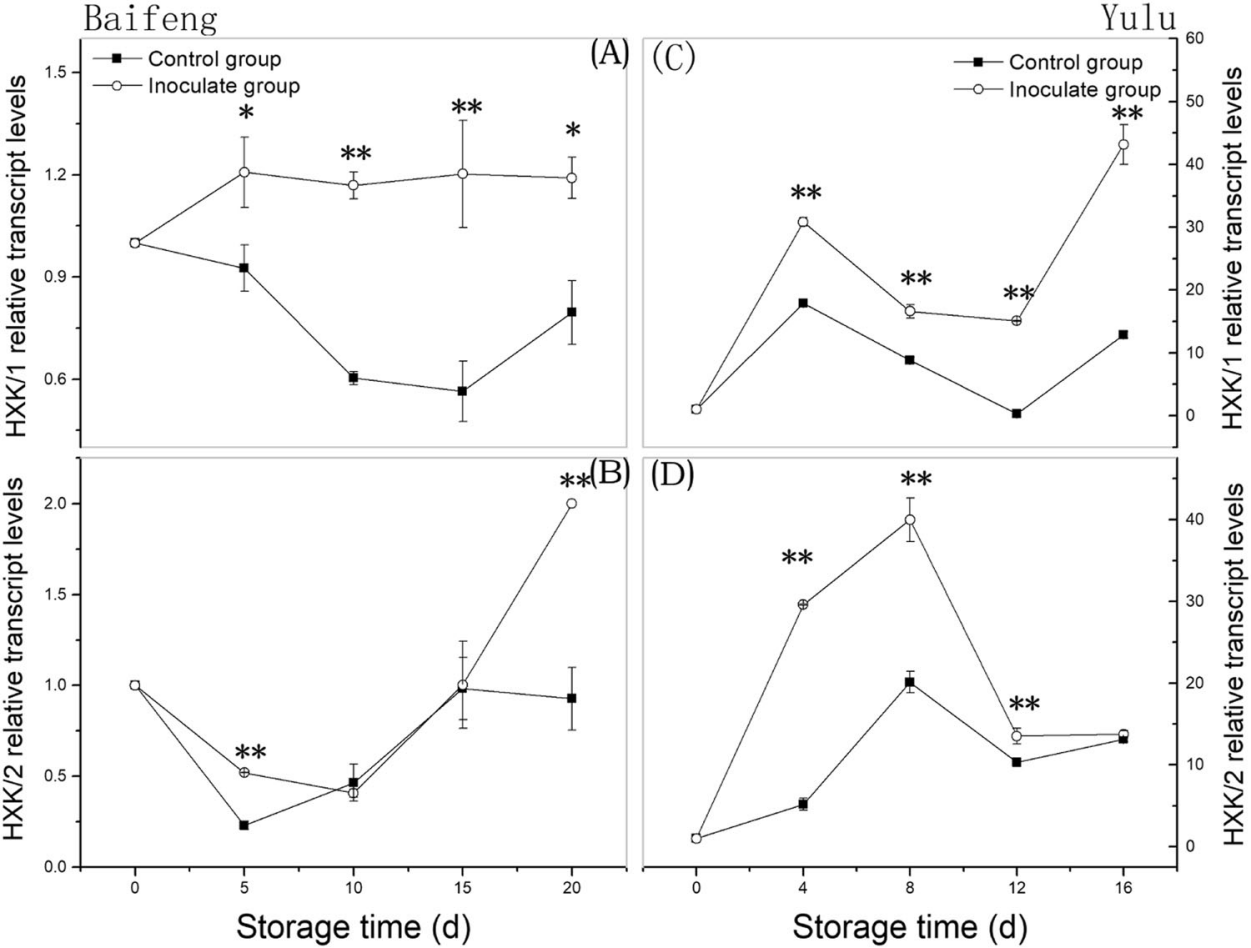
Changes in relative transcript levels for genes encoding HXK in ‘Baifeng’ (A, B) and ‘Yulu’ (C, D) peaches stored at 10 °C for 20 and 16 d, respectively. Data are expressed as the mean ± SE (*n* = 6). Asterisks indicate significant differences between control and inoculated group at each time point (*p < 0.05; **p < 0.01 based on Duncan’s multiple range test)

To determine if HXK gene expression changes as a result of fungal infection, RNA levels were measured for *HXK1* and *HXK2*. Overall, transcription for both genes appears to be enhanced in diseased fruit, although the expression profiles differ in details. Inoculated ‘Baifeng’ peaches showed a slight initial rise in *HXK1* expression, which is then maintained at a steady level (Fig. 4a). However, levels in control fruit fall, and the differences between control and diseased fruit are significant from day 5 onward. *HXK1* expression levels in inoculated and control ‘Yulu’ peaches also differ significantly across the same time period (Fig. 4c) with consistently higher levels in diseased fruit. The expression profiles for *HXK2* differ markedly between ‘Baifeng’ and ‘Yulu’ peaches. However, *HXK2* levels in diseased ‘Baifeng’ fruit were significantly (*p* < 0.05) higher than in controls on days 5 and 20, and for ‘Yulu’ peaches, levels were higher in diseased fruit except at the last time point. Thus, fungal disease increases the relative expression levels of *HXK1* and *HXK2*.

## Discussion

Peach fruit of ‘Baifeng’ and ‘Yulu’ inoculated with *M. fructicola* develop severe decay during storage at 10 °C. Our results show that as disease progresses, the sucrose content in both cultivars decreases rapidly, while reducing sugars increase. This finding agreed with the previous report on peach leaf, which showed that the diseased leaves possessed higher contents of fructose and glucose, but lowered contents of sucrose^9^. Different plants with fungal infection show different effect on sugar metabolism^19^. In tomato leaves when inoculated with *Botrytis cinerea*, both sucrose and hexose content decreased, but at the same time the hexose to sucrose ration raised^20^. The decline in soluble sugars was also observed in sunflowers during fungal infection of *Sclerotinia sclerotiorum*^21^. Tobacco leaves infected *Phytophthora. nicotianae* resulted in an increase in the levels of sucrose and hexose content^22^. Our previous report demonstrated that sucrose decomposition also occurs in peaches subjected to chilling stress^4^. Therefore, biotic (fungal disease) and abiotic (chilling injury) stress are both accompanied by rapid declines in sucrose content.

Sugar is a raw material for energy metabolism and compound synthesis in plants. It also functions as a signal molecule, in concert with hormones, nitrogen, and other signals, to regulate metabolism in plants^23^. Among soluble sugars in plants, the metabolism, partitioning, and sensing of sucrose is vital at all stages of the lifecycle^24^. Sucrose and its cleavage products are important metabolic signals that affect the expression of different gene classes, and are involved in developmental regulation^25^. Post-harvest strawberry fruit treated with β-aminobutyric acid enhanced the resistance of *B. cinerea* infection, and exhibited higher levels of sucrose, fructose and glucose, due to its higher SS-synthesis and SPS activities, and lower SS-cleavage activity^26^. Applying 0.1 or 1 mmol/L benzothiadiazole in grape cells also altered soluble sugar metabolism by increasing SS-cleavage activity, and decreasing SS-synthesis and SPS activities, resulting in lower content of glucose and sucrose, but enhanced defense-related gene expression and phytoalexin accumulation^7^. In *Arabidopsis thaliana*, AI activity increases significantly in tissues infected with *Albugo Candida*^27^. In tomato leaves, the infection of *B. cinerea* results in increasing activity of vacuole and extracellular invertases^28^. Our results show that fungal disease increases AI, NI, and SS-cleavage activities and decreases SS-synthesis and SPS activities, which accelerates sucrose decomposition and the accumulation of reducing sugars. There are also evidences suggesting that plants establish high hexose levels in response to invading pathogens, which in turn support defense responses of the host^29^. In fact in plant sucrose to hexose seems to be an important factor to defense pathogen^30^. In sunflowers infected by *S. sclerotiorum*, the expression of two hexose transporters was enhanced during infection, which indicated that fungal infection promoted hexose metabolism^21^. Fungal disease also increases the SSC in peach fruit, which agrees with the previous report in diseased mango fruit^31^.

Glucose is a major nutrient for pathogenic fungi^32^. Sucrose must first be hydrolyzed to generate hexoses, which are then imported by the fungus through a hexose transporter^33^. The EMP pathway and the pentose phosphate pathway (PPP) are the major routes for hexose oxidation. EMP is a common hexose degradation pathway in all organisms and is important for the acquisition of energy^34^. HXK, which catalyzes the phosphorylation of glucose, is the entry point for hexose in the EMP^35^. It is also a signal receptor for glucose and can regulate growth, development, and senescence in plants^36^. Pyrophosphate-dependent PFK is the primary phosphoryl donor and, like HXK, can catalyze the transfer of phosphate to the 1-position of fructose-6-phosphate^37^. This enzyme also senses energy requirement and affects the resistance to stress in plants^38^. PK is the last irreversible reaction in the EMP pathway, and plays an important role^39^. A shift in primary carbon metabolism is first response to oxidative stress. It is induced within seconds, preceding transcriptional regulation by a considerable time. The PPP produces reducing equivalents in the form of NADPH^40^. G6PDH is the first irreversible key enzyme of the PPP and catalyzes the phosphorylation of fructose^41,42^.

In this study, fungal infection enhanced PFK, HXK, and PK activities in two peach cultivars but had no significant effect on G6PDH. This implies that peaches inoculated with fungi increase their energy supply by decomposing sucrose to yield hexose, which is channeled through HXK, PK, and PFK into EMP. HXK can respond to biotic and abiotic stress in plants^43–45^. In *Arabidopsis*, the expression of *HXK2* responses to cold and salt stress^44^. Overexpression of *HXK1* and *HXK2* in *Arabidopsis* can improve resistance to pathogens^43^. In this study, fungal infection was also found to cause high expression of *HXK*.

In summary, as fungal disease progresses in peach fruit, sucrose decomposition accelerates, and provides increased levels of reducing sugars, especially glucose. Glucose acts as a substrate and, under the action of the key enzymes HXK, PK, and PFK, mobilizes the EMP pathway to resist pathogen invasion.

## References

[1] Keunen E, Peshev D, Vangronsveld J, Ende W, Cuypers A (2013). Plant sugars are crucial players in the oxidative challenge during abiotic stress: extending the traditional concept. Plant Cell Environ..

[2] Ma Y, Zhang Y, Lu J, Shao H (2010). Roles of plant soluble sugars and their responses to plant cold stress. Afr. J. Biotechnol..

[3] Anil KG, Narinder K (2005). Sugar signaling and gene expression in relation in relation to carbohydrate metabolism under abiotic stresses in plants. J. Biosci..

[4] Wang K (2013). The metabolism of soluble carbohydrates related to chilling injury in peach fruit exposed to cold stress. Postharvest Biol. Technol..

[5] Wang Z, Cao J, Jiang W (2016). Changes in sugar metabolism caused by exogenous oxalic acid related to chilling tolerance of apricot fruit. Postharvest Biol. Technol..

[6] Shao X, Zhu Y, Cao S, Wang H, Song Y (2013). Soluble sugar content and metabolism as related to the heat-induced chilling tolerance of loquat fruit during cold storage. Food Bioprocess Technol..

[7] Wang K, Liao Y, Cao S, Di H, Zheng Y (2015). Effects of benzothiadiazole on disease resistance and soluble sugar accumulation in grape berries and its possible cellular mechanisms involved. Postharvest Biol. Technol..

[8] Salzman RA, Tikhonova I, Bordelon BP, Hasegawa PM, Bressan RA (1998). Coordinate accumulation of antifungal proteins and hexoses constitutes a developmentally controlled defense response during fruit ripening in grape. Plant Physiol..

[9] Moscatello S (2017). Peach leaf curl disease shifts sugar metabolism in severely infected leaves from source to sink. Plant Physiol. Bioch..

[10] Guijarro B, Melgarejo P, De CA (2007). Effect of stabilizers on the shelf-life of *Penicillium* frequentans conidia and their efficacy as a biological agent against peach brown rot. Int. J. Food Microbiol..

[11] Koch KE (1996). Carbohydrate-modulated gene expression in plants. Annu. Rev. Plant. Physiol. Plant. Mol. Biol..

[12] Borsani J (2009). Carbon metabolism of peach fruit after harvest: changes in enzymes involved in organic acid and sugar level modifications. J. Exp. Bot..

[13] Miron D, Schaffer AA (1991). Sucrose phosphate synthase, sucrose synthase, and invertase activities in developing fruit of *Lycopersicon esculentum* mill. and the sucrose accumulating *Lycopersicon hirsutum* Humb. and Bonpl. Plant Physiol..

[14] Guo J, Jermyn WA, Turnbull MH (2002). Carbon assimilation, partitioning and export in mature cladophylls of two asparagus (*Asparagus officinalis*) cultivars with contrasting yield. Physiol. Plant..

[15] Yu F, Ni Z, Shao X, Yu L, Liu H (2015). Differences in sucrose metabolism in peach fruit stored at chilling stress versus nonchilling stress temperatures. Hortic. Sci..

[16] Yu L (2016). Effects of hot air and methyl jasmonate treatment on the metabolism of soluble sugars in peach fruit during cold storage. Postharvest Biol. Technol..

[17] Yu L, Shao X, Wei Y, Xu F, Wang H (2017). Sucrose degradation is regulated by 1-methycyclopropene treatment and is related to chilling tolerance in two peach cultivars. Postharvest Biol. Technol..

[18] Livak KJ, Schmittgen TD (2001). Analysis of relative gene expression data using real-time quantitative PCR and the 2^−ΔΔCt^ method. Methods.

[19] Berger S, Sinha AK, Roitsch T (2007). Plant physiology meets phytopathology: plant primary metabolism and plant pathogen interactions. J. Exp. Bot..

[20] Berger S (2004). Complex regulation of gene expression, photosynthesis and sugar levels by pathogen infection in tomato. Physiol. Plant..

[21] Jobic C (2007). Metabolic processes and carbon nutrient exchanges between host and pathogen sustain the disease development during sunflower infection by *Sclerotinia sclerotiorum*. Planta.

[22] Scharte J, Sch NH, Weis E (2005). Photosynthesis and carbohydrate metabolism in tobacco leaves during an incompatible interaction with *Phytophthora nicotianae*. Plant Cell Environ..

[23] León P, Sheen J (2003). Sugar and hormone connections. Trends Plant Sci..

[24] Roitsch T, González MC (2004). Function and regulation of plant invertases: sweet sensations. Trends Plant Sci..

[25] Rolland F, Moore B, Sheen J (2002). Sugar sensing and signaling in plants. Plant Cell.

[26] Wang K (2016). Induction of direct or priming resistance against *Botrytis cinerea* in strawberries by β-aminobutyric acid and their effects on sucrose metabolism. J. Agric. Food Chem..

[27] Tang X, Rolfe SA, Scholes JD (1996). The effect of *Albugo Candida* (white blister rust) on the photosynthetic and carbohydrate metabolism of leaves of *Arabidopsis thaliana*. Plant Cell Environ..

[28] Tae KH, Seung HE, Rim Y, Kim J (2011). Alteration of the expression and activation of tomato invertases during *Botrytis cinerea* infection. Plant Omics..

[29] Sen YS, Cho J, Lee SK (2007). Current insights into the primary carbon metabolic flux that occurs in plants undergoing a defense response. Plant Stress.

[30] Essmann J (2008). RNA interference-mediated repression of cell wall invertase impairs defense in source leaves of tobacco. Plant Physiol..

[31] Hu M (2014). Reduction of postharvest anthracnose and enhancement of disease resistance in ripening mango fruit by nitric oxide treatment. Postharvest Biol. Technol..

[32] Solomon PS, Tan KC, Oliver RP (2003). The nutrient supply of pathogenic fungi; a fertile field for study. Mol. Plant Pathol..

[33] Voegele RT, Mendgen KW (2011). Nutrient uptake in rust fungi: how sweet is parasitic life?. Euphytica.

[34] Desantis D, Tryon VV, Pollack JD (1989). Metabolism of mollicutes: the Embden–Meyerhof–Parnas pathway and the hexose monophosphate shunt. J. Gen. Microbiol..

[35] Petreikov M, Dai N, Granot D, Schaffer AA (2001). Characterization of native and yeast-expressed tomato fruit fructokinase enzymes. Phytochemistry.

[36] Moore B, Sheen J (2003). Role of the *Arabidopsis* glucose sensor HXK1 in nutrient, light, and hormonal signaling. Science.

[37] Couee I, Sulmon C, Gouesbet G, Amrani AE (2006). Involvement of soluble sugars in reactive oxygen species balance and responses to oxidative stress in plants. J. Exp. Bot..

[38] Ronimus RS, Morgan HW (2001). The biochemical properties and phylogenies of phosphofructokinases from extremophiles. Extremophiles.

[39] Gupta VK, Singh R (1991). Pyruvate kinase in plants—a review. Plant Physiol. Biochem..

[40] Krüger A (2011). The pentose phosphate pathway is a metabolic redox sensor and regulates transcription during the antioxidant response. Antioxid. Redox Signal..

[41] Liu J, Wang X, Hu Y, Hu W, Bi Y (2013). Glucose-6-phosphate dehydrogenase plays a pivotal role in tolerance to drought stress in soybean roots. Plant Cell Rep..

[42] Sagisaka S (1972). Decrease of glucose 6-Phosphate and 6-Phosphogluconate dehydrogenase activities in the xylem of *Populus gelrica* on budding. Plant Physiol..

[43] Sarowar S, Lee JY, Ahn ER, Pai HS (2008). A role of hexokinases in plant resistance to oxidative stress and pathogen infection. J. Plant Biol..

[44] Kreps JA (2002). Transcriptome changes for Arabidopsis in response to salt, osmotic, and cold stress. Plant Physiol..

[45] Wang X, Li L, Yang P, Gong C (2014). The role of hexokinases from grape berries (*Vitis vinifera L*.) in regulating the expression of cell wall invertase and sucrose synthase genes. Plant Cell Rep..

